# Development of theory-based health messages: three-phase programme of formative research

**DOI:** 10.1093/heapro/dau005

**Published:** 2014-02-05

**Authors:** Tracy Epton, Paul Norman, Peter Harris, Thomas Webb, F. Alexandra Snowsill, Paschal Sheeran

**Affiliations:** 1 Department of Psychology, University of Sheffield, Western Bank, Sheffield S10 2TP, UK; 2 School of Psychology, University of Sussex, Falmer BN1 9QH, UK; 3 Psychology Department, University of North Carolina, 323 Davie Hall, Chapel Hill, NC 27599-3270, USA

**Keywords:** health, theory, intervention, theory of planned behaviour

## Abstract

Online health behaviour interventions have great potential but their effectiveness may be hindered by a lack of formative and theoretical work. This paper describes the process of formative research to develop theoretically and empirically based health messages that are culturally relevant and can be used in an online intervention to promote healthy lifestyle behaviours among new university students. Drawing on the Theory of Planned Behaviour, a three-phase programme of formative research was conducted with prospective and current undergraduate students to identify (i) modal salient beliefs (the most commonly held beliefs) about fruit and vegetable intake, physical activity, binge drinking and smoking, (ii) which beliefs predicted intentions/behaviour and (iii) reasons underlying each of the beliefs that could be targeted in health messages. Phase 1, conducted with 96 pre-university college students, elicited 56 beliefs about the behaviours. Phase 2, conducted with 3026 incoming university students, identified 32 of these beliefs that predicted intentions/behaviour. Phase 3, conducted with 627 current university students, elicited 102 reasons underlying the 32 beliefs to be used to construct health messages to bolster or challenge these beliefs. The three-phase programme of formative research provides researchers with an example of how to develop health messages with a strong theoretical- and empirical base for use in health behaviour change interventions.

## INTRODUCTION

Too few young people engage in health behaviours that are known to reduce the risk of developing serious diseases and conditions including cancer, heart and circulatory disease, obesity and type 2 diabetes (Parliamentary Office of Science and Technology, 2007; National Centre for Social Research, 2008). For example, a recent national survey in England found that only 20% of young people (aged 16–24) eat five portions of fruit and vegetables daily, less than 50% meet weekly physical activity guidelines, 40% exceed daily recommended alcohol limits and 25% smoke (National Centre for Social Research, 2008). Interventions are therefore needed to promote healthy eating, physical activity and sensible alcohol consumption and to discourage smoking in young people.

Medical Research Council guidelines for the development of complex interventions emphasize the importance of conducting formative and theoretical work before proceeding to randomised controlled trials (Craig *et al.*, 2008). Formative research ensures the development of ‘targeted, culturally appropriate, health messages that work’ [(Witte *et al.*, 2001), p. 49]. The use of theory in the development of health behaviour interventions ensures that appropriate targets for intervention are identified (Michie and Prestwich, 2010). Moreover, theory-based health behaviour interventions have been found to be more efficacious (Noar *et al.*, 2007; Webb *et al.*, 2010). With the importance of theory-driven, formative research in mind, this paper reports a three-phase programme of research, based on the Theory of Planned Behaviour (TPB) (Ajzen, 1988), to develop the content of messages designed to promote four key health behaviours.

The TPB is one of the most widely applied models of health behaviour and provides clear guidelines for the identification of beliefs to target in health behaviour change interventions (Conner and Norman, 2005). According to the TPB, the proximal determinant of behaviour is intention which, in turn, is determined by (i) attitude (i.e. positive or negative evaluations of performing the behaviour), (ii) subjective norm (i.e. perceived social pressure to perform or not perform the behaviour) and (iii) perceived behavioural control (Ajzen, 1988). The TPB has been found to be predictive of health behaviour; for example, a recent meta-analysis (McEachan *et al.*, 2011) found that the TPB explained large amounts of variance in students' intentions in relation to dietary behaviours (50.3%), physical activity (52.1%) and abstinence from smoking and binge drinking (43.6%). The TPB was also found to explain significant proportions of variance in students’ prospective health behaviour for diet (21.2%), physical activity (29.7%) and abstinence (15.5%). Overall, these findings suggest that the TPB provides a strong theoretical basis for health behaviour interventions.

Health behaviour interventions are often delivered as health messages that typically comprise a number of techniques to change behaviour, such as providing information about health consequences, others’ approval and environmental cues (Michie *et al.*, 2013). Regardless of the specific techniques used in order to change health behaviour it is necessary for health messages to target the key motivational factors that underlie the behaviour (Conner and Norman, 2005). According to the TPB, interventions should target (i) behavioural beliefs about the perceived consequences of performing the behaviour, (ii) normative beliefs about the views of others and (iii) control beliefs about the power of factors to facilitate or inhibit performance of the behaviour (Ajzen, 2013). As well as its success in predicting behaviour, the TPB has also been used to develop successful interventions designed to promote health behaviour including fruit and vegetable consumption, physical activity and alcohol consumption (Jemmott *et al.*, 2011; Koning *et al.*, 2011), although effect sizes have typically been small (Hardeman *et al.*, 2002). Nevertheless, a review of early TPB-based interventions noted that many interventions did not explicitly target the TPB constructs and/or underlying beliefs in the intervention, instead the TPB was more often used to inform the choice of dependent variables to evaluate the interventions (Hardeman *et al.*, 2002). More recent health behaviour interventions based on the TPB, including those delivered online, have reported medium-sized effects on behaviour (Webb *et al.*, 2010).

Ajzen (Ajzen, 2013) proposes that extensive formative research should be conducted when developing interventions based on the TPB. This formative research should involve (i) eliciting modal salient beliefs (i.e. the most commonly held beliefs about the behaviour) and (ii) assessing which beliefs are associated with intention and/or behaviour (Sutton, 2002). The formative research is therefore used to identify which beliefs should be targeted in an intervention, typically through the development of persuasive messages. However, as noted by Eagly and Chaiken [(Eagly and Chaiken, 1998), p. 240] ‘the model provides no formal guidance for choosing arguments to include in messages designed to influence a specific belief’. Given the importance of identifying key beliefs for developing health interventions and the potential knowledge gap between those developing the intervention and the intended recipients (e.g. do academic researchers know why students binge drink?), it is surprising that these formative procedures are rarely, if ever, undertaken when developing health behaviour change interventions based on the TPB. Many TPB studies have examined the behavioural, normative and control beliefs associated with a range of health behaviours including eating fruit and vegetables, physical activity, binge drinking and smoking (e.g. Rhodes *et al.*, 2009; Murnaghan *et al.*, 2010; French and Cooke, 2012); however, these beliefs are rarely used as the basis of subsequent interventions. For example, none of nine studies included in a recent meta-analysis of online health behaviour change interventions that were based on the TPB (Webb *et al.*, 2010) used recommended formative research procedures (Sutton, 2002) to identify beliefs to target in the intervention.

### The present research

We conducted a three-phase programme of formative research to develop theory- and empirically based messages to target four important health behaviours (fruit and vegetable intake, physical activity, binge drinking and smoking) among new university students. Phase 1 identified the modal salient behavioural, normative and control beliefs that underpin pre-university students’ attitudes, subjective norms and perceived behavioural control concerning the four health behaviours. Phase 2 identified the specific beliefs that were associated with new students’ intentions to perform each of the health behaviours before university and their subsequent behaviour at university. Phase 3 identified the modal salient reasons underpinning each of the beliefs for each of the behaviours. These reasons were used as the basis for developing messages to promote regular exercise and fruit and vegetable intake and discourage excessive alcohol consumption and smoking in new university students.

## PHASE 1: BELIEF ELICITATION

### Method

Participants were pre-university students in their final term at a Further Education College in the North of England (*n* = 96). Participants were randomly assigned to complete one of four questionnaires in a classroom group setting. Each questionnaire was designed to elicit participants’ beliefs about engaging in one of four health behaviours at university: (i) eating five portions of fruit and vegetable daily (*n* = 21), (ii) engaging in regular exercise (*n* = 24), (iii) binge drinking (*n* = 26) and (iv) smoking (*n* = 25).

Behavioural beliefs were assessed by four questions for each health behaviour, e.g. ‘What do you believe would be the advantages of eating five portions of fruit and vegetables a day at university?’, ‘What do you believe would be the disadvantages of eating five portions of fruit and vegetables a day at university?’, ‘What would you like or enjoy about eating five portions of fruit and vegetables a day at university?’, and ‘What would you dislike or hate about eating five portions of fruit and vegetables a day at university?’. Normative beliefs were assessed by two questions for each health behaviour, e.g. ‘Which individuals or groups of people would approve (i.e. think it was a good idea) of you eating five portions of fruit and vegetables a day at university?’ and ‘Which individuals or groups of people would disapprove (i.e. think it was a bad idea) of you eating five portions of fruit and vegetables a day at university?’. Control beliefs were accessed by two questions for each health behaviour, e.g. ‘What things (i.e. factors or circumstances) would make you more likely to engage in eating five portions of fruit and vegetables a day at university?’ and ‘What things (i.e. factors or circumstances) would make you less likely to engage in eating five portions of fruit and vegetables a day at university?’.

### Results

Two independent raters coded all beliefs (90–92% agreement). Modal salient beliefs were defined as those mentioned by at least 20% of participants (see Tables 1–4). Five behavioural (e.g. fruit and vegetables are boring), two normative (e.g. parents approve of eating fruit and vegetables) and one control (e.g. fruit and vegetables are expensive) modal salient beliefs were identified for fruit and vegetable intake; seven, three and five behavioural, normative and control modal salient beliefs were identified, respectively, for physical activity; eight, four and four behavioural, normative and control modal salient beliefs were identified for binge drinking and seven, six and four behavioural, normative and control modal salient beliefs were identified for smoking. These beliefs were used in Phase 2 to identify the key beliefs that predict intention and/or behaviour for each health behaviour.

**Table 1:** Fruit and vegetables: summary of results from Phases 1, 2 and 3

	Phase 1: Belief elicitation		Phase 2: Behaviour prediction (*β*s)		Phase 3: Reasons elicitation		
*n*		21	702	244	117		
	Belief category		Intentions	Behaviour			
Behavioural belief	Advantages/like/enjoy	Healthy (*n* = 18)	0.13***	0.06	Important health benefits of eating fruit and vegetables	Reduced risk of disease (*n* = 68) Vitamins and minerals (*n* = 46) Boost immune system (*n* = 37) Healthy weight (*n* = 28)	
		Study better (*n* = 6)	0.24***	0.04			
		Better/varied diet (*n* = 6)	−0.02	0.06			
					How fruit and vegetables can help you study better	Energy (*n* = 48) Improved concentration (*n* = 35) Not being too ill to study (*n* = 24) Improved well-being (*n* = 22) No sluggishness (*n* = 20)	
	Disadvantages/dislike/hate	Cost (*n* = 11)	−0.15***	−0.11	Ways of eating fruit and vegetables cheaply	Buy from market/greengrocers (*n* = 56) Special offers (*n* = 31) Buy frozen (*n* = 22)	
		Boring (*n* = 6)	−0.29***	−0.23***			
					Ways to make eating fruit and vegetables interesting	Try new fruit and vegetables (*n* = 25) Try new recipes (*n* = 22) Add to dishes (*n* = 16)	
Normative beliefs	People who would approve	Parents (*n* = 7)	−0.19***	−0.06	Reasons your parents want you to eat fruit and vegetables	Health (*n* = 75) Healthy weight (*n* = 24) Helps with studies (*n* = 17)	
	People who would disapprove	Friends (*n* = 5)	−0.19***	−0.13	Reasons your friends want you to eat fruit and vegetables	Health (*n* = 82) Energy (*n* = 26) Healthy weight (*n* = 16)	
Control beliefs	Factors that would make it more likely						
	Factors that would make it less likely	Cost (*n* = 9)	0.08*	−0.02	Cost	See above

**Table 2:** Physical activity: summary of results from Phases 1, 2 and 3

	Phase 1: Belief elicitation		Phase 2: Behaviour prediction (*β*s)		Phase 3: Reasons elicitation		
*n*		24	766	270	112		
	Belief category		Intentions	Behaviour			
Behavioural belief	Advantages/like/enjoy	Health (*n* = 18)	0.16***	0.11	The important health benefits of exercise	Reducing the risk of disease (*n* = 37) Improving well-being (*n* = 18) Maintaining a healthy weight (*n* = 17)	
		Fitness (*n* = 14)	0.16***	0.08			
		Make friends (*n* = 11)	0.002	0.10			
		Socializing (*n* = 10)	0.09	−0.07			
		Stress relief (*n* = 9)	0.24***	0.07			
					The important health benefits of improving fitness	Reducing the risk of disease (*n* = 33) Increased energy (*n* = 19) Living longer (*n* = 11)	
					How exercise relieves stress	Distraction (*n* = 34) Expending negative energy (*n* = 19) Improving well-being (*n* = 11)	
	Disadvantages/dislike/hate	Lack of time for study (*n* = 16)	−0.13***	−0.07	Why/how exercise might not reduce time for study	Plan it into your day (*n* = 24) Aids concentration (*n* = 18) More productive (*n* = 12)	
		Time consuming (*n* = 16)	−0.07	0.05			
Normative beliefs	People who would approve	Sporty people (*n* = 9)	0.05	−0.05	Reasons that your parents and family think you should exercise	Health (*n* = 42) Improving well-being (*n* = 11) Helps to avoid unhealthy things (*n* = 9)	
		Family (*n* = 5)	0.10*	0.06			
		Friends (*n* = 7)	0.23***	0.11			
					Reasons that your friends think you should exercise	Health (*n* = 35) Do it together (*n* = 32) Improving well-being (*n* = 6)	
	People who would disapprove						
Control beliefs	Factors that would make it more likely	Access to range of facilities (*n* = 9)	0.23***	0.11	The best sports facilities in Sheffield	The Peak District (*n* = 15) S10 gym (*n* = 9) University swimming pool (*n* = 7)	
		Cost (*n* = 6)	0.32***	0.14			
					How to exercise for free in Sheffield	Running (*n* = 31) Cycling (*n* = 12) The Peak District (*n* = 9)	
	Factors that would make it less likely	Time restrictions (*n* = 7)	0.08*	−0.22	See above		
		Cost (*n* = 6)	0.23***	0.21			
		Poor/few facilities (*n* = 6)	−0.03	0.01

**Table 3:** Binge drinking: summary of results from Phases 1, 2 and 3

	Phase 1: Belief elicitation		Phase 2: Behaviour prediction (*β*s)		Phase 3: Reasons elicitation	
*n*		26	726	325	137 (drinkers only)	
	Belief category		Intentions	Behaviour		
Behavioural belief	Advantages/like/enjoy	Make friends/meet people (*n* = 19)	0.09*	0.20*	Ways to make friends and fun things to do at university that doesn't involve binge drinking	Socialize (e.g. cinema, eat out) (*n* = 83) Sports (*n* = 75) Join societies (*n* = 41)
		Socializing (*n* = 17)	0.06	−0.16		
		Fun *(n* = 10)	0.52***	0.38***		
		Stress relief (*n* = 7)	0.01	0.05		
	Disadvantages/dislike/hate	Impact on studies (*n* = 16)	−0.18***	−0.17*	How binge drinking has a negative impact on studies	Hangovers (*n* = 107) Missing lectures (*n* = 82) Study problems (*n* = 49)
		Hangover (*n* = 14)	0.07**	−0.01		
		Impact on health (*n* = 12)	−0.03	0.06		
		Cost (*n* = 10)	−0.01	0.001		
Normative beliefs	People who would approve	Students *(n* = 8)	0.09	0.06	Reasons your friends might not	Health (*n* = 62) Friends would have to look after you (*n* = 55) Spoil their night (*n* = 33)
		Friends *(n* = 8)	0.51***	0.30***	Want you to binge drink	
	People who would disapprove	Teachers/lecturers *(n* = 17)	0.01	−0.10	Reasons your parents think that you shouldn't binge drink	Health (*n* = 101) Bad for studies (*n* = 78) Safety (*n* = 78) Money (*n* = 50)
		Parents *(n* = 11)	0.23***	0.18*		
Control beliefs	Factors that would make it more likely	Friends drinking (*n* = 12)	0.44***	0.35***	Reasons not to binge drink even when your friends are	It's my decision (*n* = 66) Money (*n* = 59) Avoid hangover (*n* = 39) Look after friends (*n* = 30)
		Other people drinking/norms (*n* = 9)	0.23***	0.15*		
	Factors that would make it less likely	Cost *(n* = 8)	0.19***	−0.09	Reasons why they should not be influenced when they see others drinking	Their decision (*n* = 89) Poor role models (*n* = 59)
		Studies *(n* = 7)	−0.07*	0.06

**Table 4:** Smoking: summary of results from Phases 1, 2 and 3

Phase 1: Belief elicitation			Phase 2: Behaviour prediction (βs)				Phase 3: Reasons elicitation					
*N*		25	606	27	114	79	179		35		47	
			Intentions			Behaviour						
	Belief category		NS	EX	SMO	SMO	NS		EX		SMO	
Behavioural belief	Advantages/like/enjoy	Make new friends (*n* = 11)	−0.02	−0.00	−0.13	0.11	Ways of relieving stress at university	Exercise (*n* = 86) Talking things over (*n* = 28) Relaxation/meditation (*n* = 27)			How you could reduce stress without smoking	Exercise (*n* = 22) Socializing (*n* = 6) Relaxation/mediation (*n* = 5)
		Stress relief (*n* = 10)	0.12**	0.26	0.23*	−0.00						
		Socializing (*n* = 10)	0.19*	0.02	0.00	−0.34						
							Reasons why smoking doesn't help you socialize	Have to go outside (*n* = 65) The smell (*n* = 56) Puts off non-smokers (*n* = 32)				
	Disadvantages/dislike/hate	Cost (*n* = 16)	−0.12***	−0.23	−0.08	0.11	What you could spend your money on instead of smoking	Travel/holidays (*n* = 66) Nice food (*n* = 48) Socializing (*n* = 40) Clothes (*n* = 38)	Why others disapprove of smoking	Health (*n* = 19) The smell (*n* = 16) Endangers others (*n* = 9)		
		Smell (*n* = 14)	−0.01	0.18	−0.15	−0.11						
		Health risks (*n* = 11)	−0.05	−0.04	0.05	0.06						
		Others' disapproval (*n* = 8)	−0.08*	−0.51*	0.05	0.30						
							Why others disapprove of smoking	Risk to health (*n* = 85) Endangers others (*n* = 49) The smell (*n* = 63)				
Normative beliefs	People who would approve	Smokers (*n* = 16)	0.04	0.05	−0.14	−0.03						
		Friends (*n* = 8)	−0.24***	0.04	−0.01	−0.07						
	People who would disapprove	Non-smokers (*n* = 10)	−0.06	−0.37	0.14	−0.06	Why friends might not want you to smoke	Risk to health (*n* = 75) Concern about own health (*n* = 56) The smell (*n* = 55)			Why your lecturers might not want you to smoke	Health (*n* = 16) The smell (*n* = 15) Waste of study time (*n* = 14)
		Friends (*n* = 7)	−0.24***	0.04	−0.01	−0.07						
		Teachers/lecturers (*n* = 8)	−0.07	−0.17	−0.30***	0.15						
		Family/parents (*n* = 6)	−0.04	0.20	−0.33***	−0.50***						
											Why your family might not want you to smoke	Health (*n* = 30) Money (*n* = 18) The smell (*n* = 9)
Control beliefs	Factors that would make it more likely	Peer pressure (*n* = 12)	0.05	−0.40	−0.18	−0.16	How to avoid smoking when you see others smoking	Avoid smokers (*n* = 56) Think of negative aspects (*n* = 51) Remember health risks (*n* = 41) Do something else (*n* = 27)	Good ways of relieving stress other than smoking	Exercise (*n* = 18) Relax /mediation (*n* = 11) Talking it over (*n* = 6)		
		Stress (*n* = 11)	0.13**	−0.51*	0.24*	0.04						
		Other people smoking (*n* = 8)	0.17***	0.37	0.05	0.11						
							See above					
	Factors that would make it less likely	People not smoking (*n* = 10)	0.05	0.33	0.23*	0.01					Why others not smoking might reduce temptation to smoke	Don't want to smoke alone (*n* = 21) Not the norm (*n* = 6) No cues (*n* = 5)

## PHASE 2: BELIEF CORRELATION

### Method

Incoming students to a large university in the north of England (*n* = 5473) were asked by email to complete an online questionnaire 3 weeks before starting university. The students were randomly allocated to receive a link to one of four questionnaires focusing on fruit and vegetables (*n* = 1366), physical activity (*n* = 1369), binge drinking (*n* = 1370) or smoking (*n* = 1368).

The questionnaires assessed behavioural, normative and control beliefs, as well as intentions to perform the target behaviour. To measure behavioural beliefs, participants were asked to indicate (on a seven-point scale anchored by *unlikely–likely*) the extent to which they thought each of the outcomes would be likely to occur should they engage in the behaviour (e.g. ‘My eating five portions of fruit and vegetables each day at university would be healthy’). To measure normative beliefs, participants were asked to indicate (on a seven-point scale anchored by *think I should–think I should not*) the extent to which they thought specific referents would approve or disapprove of them engaging in the behaviour (e.g. my friends, my parents). To measure control beliefs, participants were asked to indicate (on a seven-point scale anchored by *less likely–more likely*) the extent to which they thought different factors would influence their behaviour (e.g. ‘The following factors would make my eating five portions of fruit and vegetables per day at university … the high cost of fruit and vegetables’). Two questions measured intentions to perform the behaviour at university on a seven-point scale (*definitely don't–definitely do*) (e.g. ‘Do you intend to eat five portions of fruit and vegetable a day at university?’).

To assess subsequent behaviour, participants were emailed a link to one of four follow-up questionnaires 1 month after starting at university that assessed their health behaviour, i.e. an eight-item fruit and vegetable intake measure (National Centre for Social Research, 2008), a three-item physical activity questionnaire (Booth, 2000), a 7-day retrospective binge drinking diary (Gmel and Rehm, 2004) or two items on smoking (Health Survey for England, 2008).

### Results

There was a 54% response rate to the baseline questionnaires (*n* = 2959): fruit and vegetables (*n* = 702), physical activity (*n* = 774), binge drinking (*n* = 735) and smoking (*n* = 747). Eighteen participants (1.12%) were excluded because they had incomplete baseline data concerning either binge drinking (*n* = 9) or physical activity (*n* = 9). There was a 39.47% response rate to the follow-up questionnaires (*n* = 1168): fruit and vegetables (*n* = 244), physical activity (*n* = 270), binge drinking (*n* = 325) and smoking (*n* = 329). For each of the four health behaviours, intentions and behaviour at follow-up were regressed separately onto behavioural beliefs, normative and control beliefs [see Tables 1–4], and intentions were correlated with subsequent behaviour.

For fruit and vegetable intake, the beliefs that predicted intention were as follows: the behavioural beliefs ‘fruit and vegetables are healthy’, ‘fruit and vegetables help you study better’, ‘fruit and vegetables are expensive’ and ‘eating fruit and vegetables is boring’; the normative beliefs ‘parents would approve of you eating fruit and vegetables’ and ‘friends would disapprove of you eating fruit and vegetables’; and the control belief ‘the cost makes eating fruit and vegetables less likely’. Intention was found to correlate significantly with subsequent fruit and vegetable intake at university, *r*(241) = 0.40, *p* < 0.001. One belief also predicted behaviour, i.e. the behavioural belief that ‘eating fruit and vegetables is boring’.

For physical activity, the beliefs that predicted intentions were: the behavioural beliefs ‘exercise is healthy’, ‘exercise improves fitness’, ‘exercise is good for stress relief’ and ‘exercise reduces time for study’; the normative beliefs ‘friends would approve of exercise’ and ‘family would approve of exercise’ and the control beliefs ‘access to a wide range of good facilities makes exercise more likely’, ‘access to cheap facilities makes exercise more likely’, ‘the cost make exercise less likely’ and ‘time restrictions makes exercise less likely’. Intention was found to correlate significantly with subsequent physical activity levels at university, *r*(264) = 0.20, *p* < 0.001. No beliefs predicted behaviour.

For binge drinking, the beliefs that predicted intentions were as follows: the behavioural beliefs ‘binge drinking is good for making friends’, ‘binge drinking is fun’, ‘binge drinking has a negative impact on studies’ and ‘binge drinking would lead to a hangover’; the normative beliefs ‘friends would approve of binge drinking’ and ‘parents would disapprove of binge drinking’ and the control beliefs ‘friends drinking makes binge drinking more likely’, ‘seeing other people drinking makes binge drinking more likely’, ‘alcohol being expensive makes binge drinking more likely’ and ‘needing time to study makes binge drinking less likely’. The same beliefs predicted behaviour, with the exception of the behavioural belief ‘hangover’ and the control beliefs ‘alcohol being expensive’ and ‘needing time to study’. Intention was found to correlate significantly with the frequency of binge drinking at university, *r*(253) = 0.64, *p* < 0.001.

Responses to the smoking survey were analysed by smoking status (non-smoker, ex-smoker and smoker). For non-smokers, the beliefs that predicted intentions to smoke were as follows: the behavioural beliefs ‘smoking is good for relieving stress’, ‘smoking is good for socializing’, ‘smoking is expensive’ and ‘smoking leads to disapproval from others’; normative beliefs ‘friends would approve of smoking’ and control beliefs ‘stress is likely to increase the temptation to smoke’ and ‘other people smoking is likely to increase the temptation to smoke’. For ex-smokers, one behavioural belief, ‘disapproval of others’ and one control belief, ‘stress makes smoking more likely’ predicted intentions to smoke. The most predictive normative belief was ‘non-smokers disapprove of smoking’. For smokers, the beliefs that predicted intentions were as follows: the behavioural belief ‘smoking relieves stress’; normative beliefs ‘your lecturers would disapprove of smoking’ and ‘your family disapprove of smoking’ and control beliefs ‘stress makes smoking more likely’ and ‘people not smoking makes smoking less likely’. Intention was found to correlate significantly with number of cigarettes smoked at university among smokers, *r*(44) = 0.29, *P* = 0.05. One normative belief ‘your family disapprove of smoking’ predicted smoking behaviour.

After removal of duplicate/similar items, 32 beliefs significantly predicted intentions: fruit and vegetable intake (6 beliefs), physical activity (8 beliefs), binge drinking (6 beliefs) and smoking (non-smokers: 6 beliefs, ex-smokers: 2 beliefs and smokers: 4 beliefs). The 32 beliefs were used in Phase 3 to elicit the reasons underlying each belief.

## PHASE 3: REASON ELICITATION

### Method

University students were invited by email to complete one of four questionnaire studies focusing on the key beliefs identified for each health behaviour: fruit and vegetables (*n* = 117), physical activity (*n* = 112), binge drinking (*n* = 137) and smoking (non-smokers *n* = 179, ex-smokers *n* = 35 and smokers *n* = 47). Participants were asked a series of questions to identify the reasons underpinning each of the 32 beliefs identified in Phase 2.

Each questionnaire asked participants to list up to three reasons underlying each belief and to indicate which of these was the most important. For example, to identify reasons underlying behavioural beliefs related to fruit and vegetable consumption participants were asked ‘*What are the three most important health benefits of eating fruit and vegetables?*’, ‘*What are the ways that fruit and vegetables can improve your studying?*’ and ‘*What are the ways you can make eating fruit and vegetables less boring?*’*.* Reasons underlying normative beliefs related to fruit and vegetable consumption were identified by asking participants ‘*What are the reasons your friends would want you to eat fruit and vegetables*’ and ‘*What are the reasons your parents would want you to eat fruit and vegetables*’. For reasons underlying control beliefs, participants were asked, ‘*What are the ways to eat fruit and vegetables cheaply*’.

### Results

Participant's responses to each of the questions were collated and coded by two independent raters (71– 100% agreement) to identify the most common reasons associated with each belief to use in health messages. The number of participants endorsing each underlying reason was calculated.

Overall, 102 reasons underlying beliefs were identified: fruit and vegetable intake (21), physical activity (24), binge drinking (19) and smoking (non-smokers: 20, ex-smokers: 6 and smokers: 12). Responses were chosen by between 5 and 107 of participants: fruit and vegetables (16–68), physical activity (9–37), binge drinking (33–101) and smoking (non-smokers 10–86; ex-smokers 6–19; smokers 5–30). For example, five reasons were identified to ‘why eating fruit and vegetables can improve your studying’: increased energy, improved concentration, no sluggishness, not being too ill to study and increased well-being. See Tables 1–4 for full details of the modal reasons for each behaviour/belief.

## DISCUSSION

A three-phase programme of formative research was conducted to identify (i) modal salient beliefs associated with four health behaviours (fruit and vegetable intake, physical activity, binge drinking, smoking), (ii) the key belief(s) that predicted intentions and/or behaviour for each health behaviour and (iii) the reasons underlying each belief. This process can be used as the basis for developing health messages that have a strong theoretical and empirical basis and that are highly relevant for the target population. The formative research reported here is in line with recommendations for good practice in the science of behavioural change such as publishing intervention content (Michie and Abraham, 2008). This enables ease of replication and allows subsequent research to build on the learning accrued during the development of the intervention, regardless of the eventual success of the intervention (Michie *et al.*, 2012). The formative research described in this paper provides a template for developing theory-based messages for health behaviour change interventions. In particular, the addition of a reasons elicitation phase provides a way of forming messages to target key beliefs identified in preceding formative research based on the TPB.

Previous attempts to develop interventions based on the TPB have been hampered by the lack of a clear procedure for constructing persuasive messages to target TPB beliefs (Eagly and Chaiken, 1998; Sutton, 2002). The reasons elicitation procedure reported in this paper provides one such procedure for developing the content of TPB persuasive messages and, in doing so, may also provide a timely impetus for the development of TPB-based interventions. Such interventions are of both practical and theoretical importance as they not only seek to change health behaviour, but also to provide important tests of theory.

There are a number of issues that warrant discussion in relation to the different phases of formative research reported in this paper. Regarding Phase 1 (i.e. belief elicitation), there are no agreed sample size guidelines for such studies. For example, studies designed to elicit the salient beliefs underlying physical activity have used samples ranging in size from 7 to 120 (Symons Downs and Hausenblas, 2005). Current recommendations for health services research (Francis *et al.*, 2004) suggest a sample size of 25, which is broadly in line with the sample sizes in the current research. A similar issue arises in relation to how to select modal salient beliefs. Ajzen and Fishbein [(Ajzen and Fishbein, 1980), p. 70] suggest that ‘those beliefs that exceed a certain frequency’ should be chosen, but fail to indicate what represents an appropriate frequency. In the present research, we selected beliefs that were endorsed by at least 20% of respondents in order to ensure that a large range of beliefs were included in Phase 2. Previous physical activity belief elicitation studies have identified a mean of seven, four and six behavioural, normative and control beliefs (Symons Downs and Hausenblas, 2005), which is similar to the numbers of beliefs chosen in the current research. However, there was some variation in the number of modal salient beliefs identified for the four health behaviours. In particular, fewer behavioural, normative and control beliefs were identified for eating fruit and vegetables than for the other health behaviours, reflecting differing levels of agreement among participants.

Regarding Phase 2 (identifying beliefs associated with intention and/or behaviour), the current research only assessed expectancy components of behavioural, normative and control beliefs, rather than testing expectancy X value combinations. According to the TPB, behavioural beliefs about the likelihood of the behaviour leading to a particular outcome should be weighted (multiplied) by the value attached to the outcome. However, only assessing expectancy components has three key advantages. First, it halves the number of items that are needed to assess the beliefs. Secondly, it avoids problems associated with the scaling and analysis of multiplicative composites (Evans, 1991). Thirdly, it produces similar, or stronger, correlations with TPB constructs as does using expectancy X value multiplicative terms (Gagne and Godin, 2000; Rhodes *et al.*, 2009).

The present study examined associations between the modal salient beliefs and intention and subsequent behaviour in new university students, in line with Sutton's (Suttons, 2002) recommendations and consistent with other TPB studies (e.g. Rhodes *et al.*, 2009; Booth *et al.*, in press). Interventions should then target these beliefs in order to produce changes in intention and/or behaviour. However, according to the TPB, behavioural, normative and control beliefs determine attitude, subjective norm and perceived behavioural control which, in turn, determine intention and behaviour. Thus, an alternative approach taken in other TPB studies (e.g. Symons Downs and Hausenblas, 2005; Murnaghan *et al.*, 2010) is to identify the modal salient beliefs that are most strongly associated with their respective constructs. Interventions should then target these beliefs in order to produce changes in attitude, subjective norm and perceived behavioural control which, in turn, should produce changes in intention and behaviour. The latter approach is more consistent with the theoretical structure of the TPB; however, given the longer proposed causal chain, any changes in underlying beliefs may not carry through to produce measurable changes in behaviour (Sutton, 2002).

It is also noteworthy that very few of the beliefs were found to be predictive of subsequent health behaviour at university, despite a larger number being predictive of intentions. As a result, most of the beliefs identified in Phase 2 to be used in Phase 3 to develop health messages were based on significant associations with intention only. However, this pattern of results is consistent with the theoretical structure of the TPB in which intention mediates the effects of attitude, subjective norm and perceived behavioural control on behaviour (although a direct link is hypothesized between perceived behavioural control and behaviour). In the current studies, the intention measures were found to have significant correlations with subsequent behaviour, suggesting that changing underlying behavioural, normative and control beliefs may impact on intentions which, in turn, impact on subsequent behaviour.

Regarding Phase 3 (reasons elicitation), as with Phase 1, issues arise regarding the number of participants needed for such studies and cut-off points for selecting modal salient reasons. In the present study the criterion for selecting modal salient reasons varied for each belief depending on the number of reasons listed and numbers of participants endorsing them, to ensure an appropriate number of reasons to be used in subsequent health messages to change the beliefs. In line with most interventions based on the TPB (for a review, see Hardeman *et al.*, 2002), the present research sought to develop messages to change existing beliefs. However, although the formative research ensured that the resultant messages were relevant for the target population, it does not guarantee that the messages will be persuasive. At present, there is a lack of research on what constitutes a ‘strong’ message (Sutton, 2002). Nonetheless, culturally relevant messages that target the key beliefs that influence behaviour, such as the ones developed using this programme of research, are likely to make strong messages.

The use of the TPB as a theoretical framework to develop health behaviour change interventions is not without its own limitations. Despite the strong predictive utility of the TPB (McEachan et al., 2011), the model has been criticized for failing to account for other, non-cognitive, influences on health behaviour (Norman and Conner, 2005). In particular, past behaviour is often found to be the strongest predictor of future behaviour suggesting the impact of habitual processes on health behaviour (Ouellette and Wood, 1998). Thus, habit strength has been found to be a strong correlate of a range of health behaviours. For example, Gardner *et al.* (Gardner *et al.*, 2011) reported a medium-to-strong average correlation (*r_+_*= 0.44) between habit strength and behaviour in their meta-analysis of 23 studies of nutrition and physical activity behaviour. This is very similar to the average intention–behaviour correlation (*r_+_* = 0.44) reported by McEachan *et al.* (McEachan *et al.*, 2011) across 237 prospective tests of the TPB in relation to health behaviour. Similarly, Norman (Norman, 2011) found that both intention and habit strength were predictive of binge drinking in university students, suggesting that interventions should focus on both intentional (e.g. underlying beliefs) and habitual (e.g. environmental cues) influences when seeking to change health behaviour.

The formative research reported in the current paper describes three phases in the development of health messages based on the TPB. However, it is important to note that there is a wide range of behaviour change techniques that can be used to promote health behaviour change in addition to the construction of health messages targeting underlying beliefs (Michie *et al.*, 2013). Moreover, a process of intervention mapping (Bartholemew *et al.*, 2011) can be used to select theory-based behaviour change techniques to target specific determinants of the targeted health behaviour. This work should then be followed by further activities focusing on the delivery, adoption/implementation and evaluation of the intervention.

## CONCLUSION

The three-phase programme of research described in this paper provides a clear, replicable procedure for developing health message that have a strong theoretical and empirical basis. The formative research includes identifying (i) the modal salient beliefs associated with eacsh health behaviour, (ii) the key beliefs associated with intentions to perform each behaviour and (iii) the key reasons/arguments to construct health messages to target each belief. This systematic process provides the basis for creating health messages that are theoretically and empirically based and highly relevant for the target population. In addition, describing the process of developing the messages promotes transparency in the reporting intervention content and thereby adds to the science of behaviour change (Michie and Abraham, 2008). The beliefs identified in this formative research can be used to form the basis of persuasive messages designed to change health behaviours in young people (Epton *et al*., 2013).

## FUNDING

The work reported in this paper was supported by the UK National Prevention Research Initiative (NPRI) Phase 4 (grant number: MR/J0004501/1). The NPRI includes the following Funding Partners (in alphabetical order): Alzheimer's Research Trust, Alzheimer's Society, Biotechnology and Biological Sciences Research Council, British Heart Foundation, Cancer Research UK, Chief Scientist Office, Scottish Government Health Directorate, Department of Health, Diabetes UK, Economic and Social Research Council, Health and Social Care Research and Development Division of the Public Health Agency (HSC and R and D Division), Medical Research Council, The Stroke Association, Wellcome Trust, Welsh Assembly Government and World Cancer Research Fund. Funding to pay the Open Access publication charges for this article was provided by the RCUK University Publication Fund at The University of Sheffield.

## References

[DAU005C1] Ajzen I. (1988) Attitudes, Personality and Behaviour. Open University Press, Milton Keynes.

[DAU005C2] Ajzen I. (2013) Behavioural interventions based on the theory of planned behaviour. http://people.umass.edu/aizen/pdf/tpb.intervention.pdf (last accessed 7 February 2013).

[DAU005C3] Ajzen I. Fishbein M. (1980) Understanding Attitudes and Predicting Social Behaviour. Prentice-Hall, Englewood Cliffs, NJ.

[DAU005C4] Bartholemew L. K. Parcel G. S. Kok G. Gottleib N. H. Fernadez M. E. (2011) Planning Health Promotion Programs: An Intervention Mapping Approach, 3rd edition Wiley, Chichester.

[DAU005C5] Booth M. L. (2000) Assessment of physical activity: an international perspective. Research Quarterly for Exercise and Sport, 71, s114–s120. 10925833

[DAU005C6] Booth A. Norman P. Goyder E. Harris P. (in press) Using the Theory of Planned Behavior to identify key beliefs underlying chlamydia testing intentions in a sample of young people living in deprived areas. Journal of Health Psychology. doi:10.1177/1359105313510335. 10.1177/1359105313510335 24287801

[DAU005C7] Conner M. Norman P. (2005) Predicting Health Behaviour: Research and Practice with Social Cognition Models, 2nd edition Open University Press, Buckingham.

[DAU005C8] Craig P. Dieppe P. Macintyre S. Michie S. Nazareth I. Petticrew M. (2008) Developing and evaluating complex interventions: the new Medical Research Council guidance. British Medical Journal, 337, 979–983. 10.1136/bmj.a1655 PMC2769032 18824488

[DAU005C9] Eagly A. H. Chaiken S. (1998) Attitude structure and function. In: Gilbert D. T. Fiske S. T. Gardner L. (eds), The Handbook of Social Psychology, 4th edition Vols 1 and 4 McGraw-Hill, New York, pp. 269–322.

[DAU005C37] Epton T. Norman P. Sheeran P. Harris P. R. Webb T. L. Ciravegna F. (2013) A theory based online health behaviour intervention for new university students: Study protocol. BMC Public Health, 13, 107. 23384237 10.1186/1471-2458-13-107 PMC3570293

[DAU005C10] Evans M. G. (1991) The problem of analyzing multiplicative composites: interactions revisited. American Psychologist, 46, 6–15.

[DAU005C11] Francis J. J. Eccles M. P. Johnston M. Walker A. Grimshaw J. Foy R. (2004) Constructing Questionnaires Based on the Theory of Planned Behaviour: A Manual for Health Services Researchers. Newcastle, UK http://pages.bangor.ac.uk/~pes004/exercise_psych/downloads/tpb_manual.pdf (last accessed 7 February 2013).

[DAU005C12] French D. P. Cooke R. (2012) Using the theory of planned behaviour to understand binge drinking: the importance of beliefs for developing interventions. British Journal of Health Psychology, 17, 1917. 10.1111/j.2044-8287.2010.02010.x 22233102

[DAU005C13] Gagne C. Godin G. (2000) The theory of planned behaviour: some measurement issues concerning belief-based variables. Journal of Applied Social Psychology, 30, 2173–2193.

[DAU005C14] Gardner B. de Bruijn G.-J. Lally P. (2011) A systematic review and meta-analysis of applications of the self-report habit index to nutrition and physical activity behaviours. Annals of Behavioral Medicine, 42, 174–187. 21626256 10.1007/s12160-011-9282-0

[DAU005C15] Gmel G. Rehm J. (2004) Measuring alcohol consumption. Contemporary Drug Problems, 31, 467–540.

[DAU005C16] Hardeman W. Johnston M. Johnston D. W. Bonetti D. Wareham N. J. Kinmonth A. L. (2002) Application of the theory of planned behaviour in behaviour change interventions: a systematic review. Psychology and Health, 17, 123–158.

[DAU005C17] Jemmott J. B. Jemmott L. S. Leary A. O. Ngwane Z. Icard L. Bellamy S. (2011) Cognitive-behavioural health-promotion intervention increases fruit and vegetable consumption and physical activity among South African adolescents. Psychology and Health, 26, 167–185. 21318928 10.1080/08870446.2011.531573 PMC4349628

[DAU005C18] Koning I. M. van den Eijnden R. J. Verdurmen J. E. Engels R. C. Vollebergh W. A (2011) Long-term effects of a parent and student intervention on alcohol use in adolescents. American Journal of Preventative Medicine, 40, 541–547. 10.1016/j.amepre.2010.12.030 21496753

[DAU005C19] McEachan R. R. C. Conner M. Taylor N. J. Lawton R. J. (2011) Prospective prediction of health-related behaviours with the theory of planned behaviour: a meta-analysis. Health Psychology Review, 5, 97–144.

[DAU005C20] Michie S. Abraham C. (2008) Advancing the science of behaviour change: a plea for scientific reporting. Addiction, 103, 1409–1410. 18783495 10.1111/j.1360-0443.2008.02291.x

[DAU005C21] Michie S. Prestwich A. (2010) Are interventions theory based? Development of an intervention theory coding scheme. Health Psychology, 29, 1–8. 20063930 10.1037/a0016939

[DAU005C22] Michie S. Brown J. Geraghty A. W. A. Miller S. Yardley L. Gardner B. (2012) Development of StopAdvisor: a theory-based interactive internet-based smoking cessation intervention. Transtheoretical Behavioural Medicine, 2, 263–275. 10.1007/s13142-012-0135-6 PMC3717907 24073123

[DAU005C23] Michie S. Richardson M. Johnston M. Abraham C. Francis J. Hardeman W. (2013) The behavior change technique taxonomy (v1) of 93 hierarchically clustered techniques: building an international consensus for the reporting of behavior change interventions. Annals of Behavioural Medicine, 46, 81–95. 10.1007/s12160-013-9486-6 23512568

[DAU005C24] Murnaghan D. A. Blanchard C. M. Rodgers W. M. LaRoas J. N. MacQuarrie C. R. MacLellan D. L. (2010) Predictors of physical activity, healthy eating and being smoke free in teens: a theory of planed behaviour approach. Psychology and Health, 8, 925–941. 20204952 10.1080/08870440902866894

[DAU005C25] National Centre for Social Research (2008). Health survey for England 2008. http://www.ic.nhs.uk/statistics-and-data-collections/health-and-lifestyles-related-surveys/health-survey-for-england (last accessed 7 February 2013).

[DAU005C26] Noar S. M. Benac C. N. Harris M. S. (2007) Does tailoring matter? Meta-analytic review of tailored print health behaviour change interventions. Psychological Bulletin, 133, 673–693. 17592961 10.1037/0033-2909.133.4.673

[DAU005C27] Norman P. (2011) The theory of planned behaviour and binge drinking among undergraduate students: assessing the impact of habit strength. Addictive Behaviors, 36, 502–507. 21310540 10.1016/j.addbeh.2011.01.025

[DAU005C28] Norman P. Conner M. (2005) Predicting and changing health behaviour: future directions. In: Conner M. Norman P. (eds), Predicting Health Behaviour: Research and Practice with Social Cognition Models, 2nd edition Open University Press: Buckingham, pp. 324–371.

[DAU005C29] Ouellette J. Wood W. (1998) Habit and intention in everyday life: the multiple processes by which past behavior predicts future behavior. Psychological Bulletin, 124, 54–74.

[DAU005C30] Parliamentary Office of Science and Technology. (2007) Health behaviour. Http://www.parliament.uk/documents/post/postpn283.pdf (last accessed 7 February 2013).

[DAU005C32] Rhodes R. E. Blanchard C. M. Courneya K. S. Plotnikoff R. C. (2009) Identifying belie-based targets for the promotion of leisure-time walking. Health Education and Behaviour, 36, 381–393. 10.1177/1090198107308376 18077658

[DAU005C33] Sutton S. (2002) Using social cognition models to develop health behaviour interventions. In: Rutter D. Quine L. (eds), Changing Health Behaviour. Open University Press: Buckingham, pp. 193–208.

[DAU005C34] Symons Downs D. Hausenblas H. A. (2005) Elicitation studies and the theory of planned behaviour: a systematic review of exercise beliefs. Psychology of Sport and Exercise, 6, 1–31.

[DAU005C35] Webb T. L. Joseph J. Yardley L. Michie S (2010) Using the Internet to promote health behaviour change. Journal of Medical Internet Research, 12:e4. 20164043 10.2196/jmir.1376 PMC2836773

[DAU005C36] Witte K. Meyer G. Martell D. (2001) Effective Health Risk Messages: A Step-by-Step Guide. Sage, Thousand Oaks, CA.

